# Depression and caries in adolescents: role of social inequities and sugar consumption

**DOI:** 10.1007/s00784-025-06738-y

**Published:** 2026-01-30

**Authors:** Ronaldo Nogueira Filho, Lorena Lúcia Costa Ladeira, Izabel Cristina Vieira de Oliveira, Luiza Jesus de Queiroz, Caroline Cabral Santos, Claudia Maria Coelho Alves, Erika Bárbara Abreu Fonseca Thomaz, Cecília Cláudia Costa Ribeiro

**Affiliations:** 1 Federal University of Maranhão, São Luís, Maranhão, Brasil; 2 Postgraduate Program of Dentistry, Federal University of Maranhão, São Luís (MA), Av. dos Portugueses 1966, Cidade Universitária Bacanga, CEP 65080-805 Brazil

**Keywords:** Adolescent health, Social class, Sugars, Dental caries, Depression

## Abstract

**Introduction:**

Caries and depression are prevalent non-communicable diseases among adolescents that may share common risk factors. This study investigated the correlation between dental caries and depression in adolescents, analyzing pathways triggered by low socioeconomic status and higher sugar consumption.

**Methods:**

This population-based sample with a cross-sectional design using data from the birth cohort at the follow-up at 18 to 19 years (*n* = 2515). *Low Socioeconomic Status* was treated as a latent variable, and sugar consumption was measured in grams (< 25 g/day). Caries diagnosis was assessed using the DMFT index, and depression using the M.I.N.I. questionnaire. The theoretical model explored caries and depression as outcomes, as well as the explanatory variables: socioeconomic status and added sugar consumption.

**Results:**

Lower socioeconomic status was associated with higher sugar consumption (CP = 0.05; *p* < 0.001) and depression (CP = 0.088; *p* = 0.013). Sugar consumption had a direct effect on depression (CP = 0.075; *p* = 0.024) and the DFMT index (CP = 0.049; *p* = 0.016). The DMFT and depression were correlated (CP = 0.068; *p* = 0.041).

**Conclusion:**

Our findings suggest an association between tooth decay and depression in adolescents, driven by shared risk factors such as lower socioeconomic status and increased sugar consumption. Implementing early intervention strategies targeting these shared risk factors is essential for mitigating the burden of chronic diseases.

**Supplementary Information:**

The online version contains supplementary material available at 10.1007/s00784-025-06738-y.

## Introduction

Adolescence is a sensitive period of human development due to the rapid physical, emotional, and social transformations that occur, as well as the establishment of behaviors that can be risk factors for the development and progression of non-communicable diseases (NCDs) [1, 2].

Among NCDs affecting adolescents, depression stands out as a major mental health concern, being the second leading cause of death among individuals aged 15 to 29 and a key contributor to the global burden of disease [3, 4]. In Brazil, data from the National Health Survey (2019) revealed a prevalence of depressive symptoms of 10.9% among young people aged 19 to 24 years [5]. Beyond its high frequency, depression substantially impairs quality of life and, in severe cases, can lead to suicide [6]. At the same time, oral conditions also represent an important public health challenge. Dental caries, in particular, remain the most prevalent disease worldwide, with an estimated prevalence of 44.5% according to the Global Burden of Disease Study 2019 [7].

People with psychological disorders are at greater risk of developing oral diseases, including dental caries, due to factors such as lifestyle, poor oral hygiene, and difficulty accessing dental care due to severe mental illness [8]. Furthermore, hyposalivation caused by the use of psychotropic drugs may contribute to poor oral health [9]. Additionally, the relationship between mental conditions and caries may be linked to common risk factors, such as social vulnerability. Social determinants linked to poverty are associated with higher rates of cavities [10], as well as an increased risk of depression and worsening psychiatric symptoms [11, 12]. Also, sugar consumption is an established risk factor for tooth decay [13] and, more recently, has been implicated in mental disorders such as anxiety, depression, and suicidal ideation [14–16].

Although adolescents are also affected by depression, this condition has been explored little in relation to dental caries. A previous study investigating this association in adolescents [17] has provided significant evidence; however, it was based on self-reported or indirect measures of depression. In this context, employing standardized diagnostic interviews may enhance the accuracy of case definition. In addition, the potential mediating role of sugar consumption in this relationship has not yet been examined, despite its well-known impact on both mental and oral health.

Considering caries and depression as NCDs that share common risk factors, we hypothesize that there is an association between these two diseases triggered by factors such as sugar consumption and socioeconomic status. Therefore, the objective of this study was to evaluate the correlation between tooth decay and depression in adolescents, analyzing the pathways influenced by poorer socioeconomic conditions and higher sugar consumption.

## Methods

### Study design and ethical considerations

This is a cross-sectional study nested within a birth cohort belonging to the RPS cohort consortium, involving follow-up with adolescents born in the city of São Luís, Maranhão, Brazil. This population-based sample is representative of adolescents in this city aged 18 and 19, born in 1997/1998.

The cohort began at birth in 10 public and private hospitals in São Luís from March 1997 to February 1998 and included 94.1% (*n* = 2,541) of all live births in the city during that period. Details of the methodology for the cohort baseline were published by Silva et al. (2001) [18].

Follow-up of the cohort during adolescence was conducted from January to December 2016 with adolescents aged 18 and 19. Participants from the initial cohort were recruited from military enlistment records, the 2014 school census, and universities, yielding 687 adolescents. To increase statistical power and ensure representativeness, the cohort was expanded via a lottery from the Brazilian Live Birth Information System (SINASC), which provided a random sample of 1,133 individuals born in São Luís in 1997. Additionally, 695 volunteers from schools and universities registered in SINASC were included.

Thus, the final sample consisted of 2,515 adolescents, combining participants from the original and expanded cohorts. This strategy preserved the population-based nature of the study and ensured that the sample was representative of adolescents born in 1997–1998 in São Luís.

The Research Ethics Committee of the Federal University of Maranhão approved the study, under the protocol no. 1,302,489, and all participants and their legal representatives signed an informed consent form. This work adhered to the principles outlined in the 1975 Declaration of Helsinki and followed the Strengthening the Reporting of Observational Studies in Epidemiology (STROBE) guidelines.

### Data collection

Data were collected from adolescents regarding their socioeconomic and demographic characteristics (gender, adolescent’s education, head of the family’s education, economic class, and monthly family income), as well as their consumption of foods rich in added sugars, and the diagnosis of depression and caries.

The consumption of added sugar was assessed using the Food Frequency Questionnaire (FFQ), which consisted of 106 types of food items adapted to the local context. Added sugar intake was estimated using a Food Frequency Questionnaire (FFQ) containing 106 items, previously validated for Brazilian adolescents [19] and adapted to reflect regional dietary habits [20]. Sugar intake estimated over the previous 12 months was used as a continuous variable in the analysis.

To quantify sugar intake, data on the consumption of various processed products containing added sugars were collected, including soft drinks, processed juices, chocolate drinks, energy drinks, sweet biscuits, dairy products, breads, breakfast cereals, chocolate, desserts, mayonnaise, snacks, and cold foods. Sugar intake was calculated by summing the daily amount in grams, based on the recommendation of the American Heart Association, which sets a maximum daily sugar consumption limit of 25 g [21]. Due to the high sugar consumption observed in the population under study, categories were created to classify intake levels: up to 25 g, 25 to 49.9 g, 50 to 74.9 g, and over 75 g.

Depression was defined based on diagnostic criteria using the Mini International Neuropsychiatric Interview Questionnaire (M.I.N.I.), a standardized diagnostic interview validated for epidemiological studies, based on the Diagnostic and Statistical Manual of Mental Disorders, Fourth Edition (DSM-IV), and the International Classification of Diseases, Tenth Revision (ICD-10) [22]. Depression was defined by the presence of either a major depressive episode or a recurrent major depressive episode, as assessed by the structured questions of the instrument.

Dental caries diagnosis was determined using the DMFT index (Decayed, Missing, and Filled Teeth) [23] according to the World Health Organization criteria [24]. A tooth was considered decayed (D) when a cavitated lesion with visible dentin involvement was present; missing (M) when it had been extracted due to caries, as reported by the participant and confirmed by clinical examination; and filled (F) when a permanent restoration was present, with or without recurrent caries. Third molars were excluded from the assessment. The clinical dental examinations were conducted by six previously calibrated examiners under artificial light in a dental unit within the research collection facilities. Before data collection, all examiners participated in theoretical and practical training sessions, followed by a calibration process that yielded an inter-examiner kappa coefficient of 0.82 and an intra-examiner coefficient of 0.74 for DMFT, indicating substantial agreement. Moreover, the visible plaque index (VPI) was evaluated on four surfaces of all teeth, excluding the third molars.

### Latent variable

The latent variable *Low Socioeconomic Situation* was constructed using the following indicators: education of the head of the family, education of the adolescent, monthly family income (based on the Brazilian national minimum wage in 2016 - R$ 880.00), and economic class (A, B, C, D, and E), as measured by the Brazilian Economic Classification Criteria [25].

### Theoretical model

**Fig. 1:**
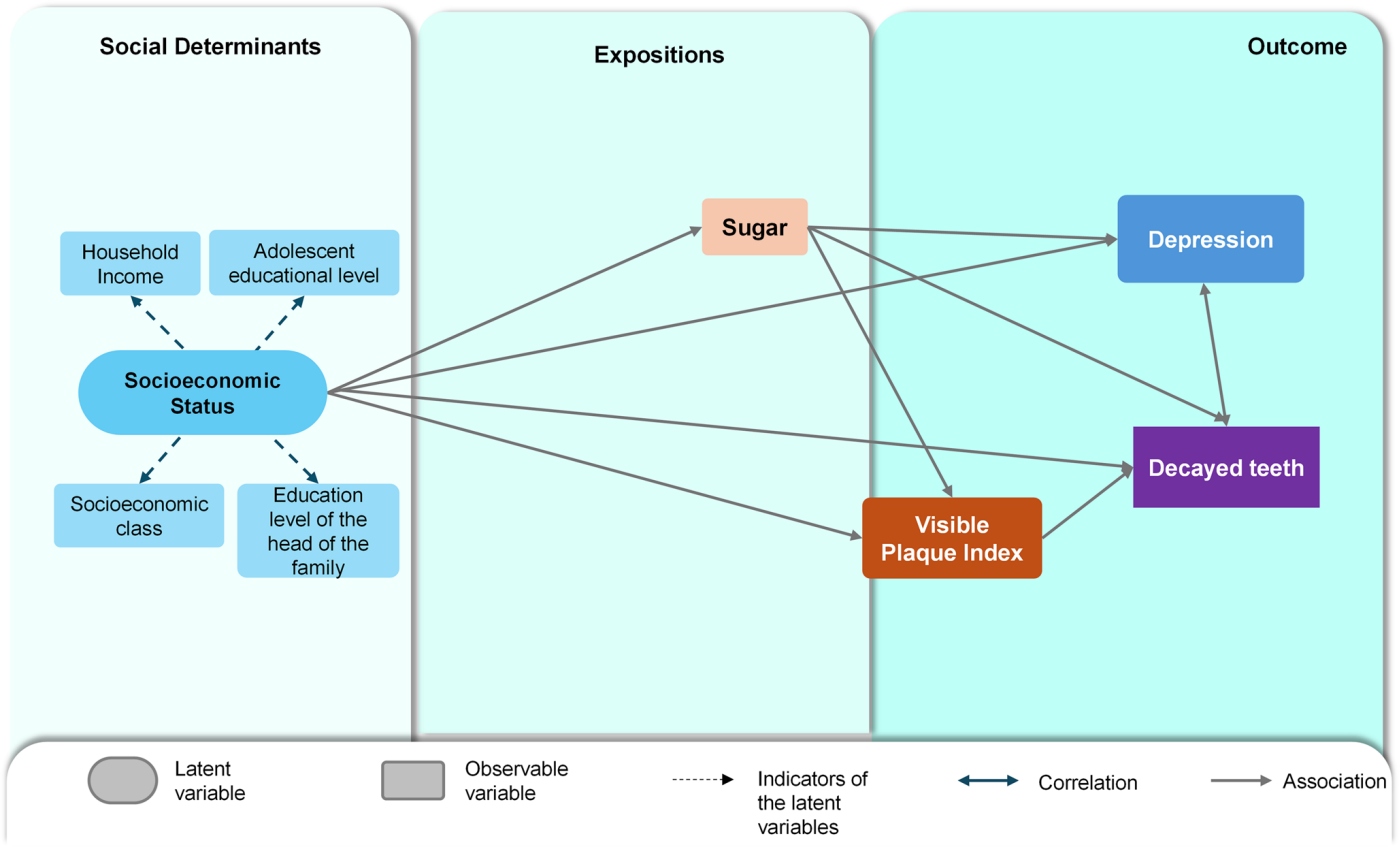
Theoretical model proposed to estimate the co-occurrence of caries and depression in adolescents. São Luís, Brazil (2023)

The model was adjusted for sex, with male coded as 1 and female as 2. In the proposed theoretical model (Fig. 1), family socioeconomic status was the most distal determinant (exogenous variable), exerting effects on the other variables in the model: sugar consumption, depression, VPI, and DMFT. A worse socioeconomic situation would increase the consumption of foods rich in added sugars, the incidence of caries lesions, and the manifestation of symptoms related to depression. Sugar consumption would have a direct influence on depression and tooth decay, and an indirect influence on tooth decay via VPI. Finally, caries and depression would be correlated with each other (double arrow).

### Statistical analysis

The proposed theoretical model was tested using Structural Equation Modeling (SEM), which allows for the identification and interpretation of direct and indirect paths between multiple exposures and outcomes, as well as the composition of latent variables and reduction of measurement errors [26].

Regarding latent variables, the following parameters were adopted: convergent factor loadings above 0.5 in Exploratory Factor Analysis (EFA); subsequently, the latent variables were confirmed using Confirmatory Factor Analysis (CFA). Mplus software version 8.0 was used, with the following criteria: a p-value greater than 0.05 in the chi-square test (χ²); a *Root Mean Square Error of Approximation* (RMSEA) *p* < 0.06 and an upper limit of the 90% confidence interval lower than 0.08; and c) *Comparative Fit Index* (CFI) and *Tucker-Lewis Index* (TLI) > 0.95. For SEM, the WLSMV estimator and theta parameterization were used. To determine whether the theoretical model had a good fit, the same criteria previously described for CFA were considered [27]. Missing data were imputed using Maximum Likelihood Estimation (MLE), assuming they were missing at random, thereby incorporating all available information without deletion, thereby preserving statistical power and reducing potential bias [28].

### Sensitivity analysis using IPTW

To comprehensively account for potential confounding, we performed a sensitivity analysis using inverse probability of treatment weighting (IPTW). Propensity scores were estimated using a logistic regression model including sex, household head’s education, household income, obesity, and the visible plaque index. Stabilized weights were calculated as the marginal probability of exposure divided by the individual propensity score and truncated at the 1 st and 99th percentiles to minimize the influence of extreme weights. Average treatment effects (ATE; risk differences) were estimated using IPTW with the teffects ipw command, employing robust variance estimators. Marginal risk ratios were additionally estimated using Poisson regression with robust standard errors weighted by the stabilized IPTW. These analyses allowed us to estimate marginal (population-averaged) effects of high sugar intake on depression and caries, complementing the multivariable adjusted regression models.

## Results

According to our findings, 52.45% of the sample comprised female individuals. Regarding socioeconomic status, it was observed that 42.9% of adolescents came from families with an income between one and three minimum wages; 69.9% had completed high school; the majority of family heads, 50.10%, had also completed secondary education, and 50.2% belonged to classes C or D. Concerning sugar intake, 32.09% consumed more than 75 g/day. According to the M.I.N.I. questionnaire, 11.8% had depression, 53.14% had an VPI greater than or equal to 15%, and the average DMFT per individual was 3.7 teeth (Table 1). A detailed descriptive distribution of sociodemographic and behavioral characteristics by depressive symptom status is provided in Supplementary Table S1.

**Table 1:** Sociodemographic data, sugar consumption, depression and DMFT of the studied population

Variables	*n*	%
Sex		
Male	1196	47.55
Female	1319	52.45
Family income, Brazilian monthly minimum wage		
< 1	797	31.69
1 a < 3	1079	42.90
3 a < 5	338	13.44
≥ 5	285	11.33
Missings	16	0.64
Adolescent’s education		
Elementary School	83	3.30
High school	1758	69.90
Faculty	672	26.72
Missings	2	0.08
Education of the head of the family		
Illiterate	286	11.37
Elementary School	563	22.39
High school	1260	50.10
Incomplete higher	81	3.22
Graduated	325	12.92
Family economy class (ABEP)		
A	94	4.22
B	565	25.37
C/D	1118	50.20
E	450	20.21
Sugar		
< 25 g	466	18.65
25 g a 49.9 g	705	28.21
50 g a 74.9 g	526	21.05
> 75 g	802	32.09
Depression		
Yes	296	11.8
No	2219	88.2
VPI		
< 15%	952	46.86
≥ 15%	1328	53.14
DMFT	Mean 3.70	SD 3.26

The final models had a satisfactory fit across all indices used: RMSEA (*p* < 0.05), RMSEA 90% confidence interval (0.027–0.042), CFI (0.982), and TLI (0.961) (Table 2).

**Table 2:** Structural equation model fit measures

Model fit indices	Expected indices	DMFT Model Indices	ISG Model Indices
***X***^2*^		83.121	85.402
Degrees of freedom		21	21
***p***value***X***^***2***^		0.0000	0.000
RMSEA ^†^	< 0.05	0.034	0.035
90% CI^‡^	< 0.08	0.027–0.042	0.027–0.043
***P*** ^§^	> 0.05	1.000	0.999
CFI ^||^	> 0.90	0.982	0.983
TLI^#^	> 0.90	0.961	0.964

^*^Chi-squared test. ^†^ Root mean square error of approximation. ^‡^ Confidence interval. ^§^p value. ^||^ Comparative fit index. ^#^Tucker Lewis index

The latent variable analyzed, socioeconomic status, showed convergent factor loadings with indicators that had a factor loading greater than or close to 0.5 and a p-value less than 0.001 (Table 3).

**Table 3:** Factor loading, standard error and p-value for the effect indicators of the syndemic situation evaluated

Latent variables	Standardized coefficients	Standard error	*p*
Socioeconomic Status			
Family income	0.606	0.019	< 0.001
Adolescent’s education	0.547	0.022	< 0.001
Occupation of the head of the family	0.661	0.017	< 0.001
Economic class	0.889	0.019	< 0.001

Regarding the standardized coefficients and p-values for total, direct, and indirect effects, socioeconomic status increased both sugar consumption (CP = 0.05; *p* < 0.001) and depression (CP = 0.088; *p* = 0.013). Sugar consumption had a direct effect on both depression (CP = 0.075; *p* = 0.024) and caries (CP = 0.049; *p* = 0.016), and an indirect effect on caries mediated by VPI (CP = 0.007; *p* = 0.016). DMFT and depression were correlated (CP = 0.068; *p* = 0.041). Additional associations were observed: female sex had an effect on sugar consumption (CP = 0.085; *p* < 0.001) and depression (CP = 0.266; *p* < 0.001) (Table 4).

**Table 4:** Standardized coefficient, standard error and p-value for the total, direct and indirect effects of the explanatory variables on the studied outcomes

Explanatory Variable	Outcome	Effect	Standardized coefficient	Standardized error	*p*
*Socioeconomic Situation*	Sugar	Direct	0.050	0.017	**< 0.001**
*Socioeconomic Situation*	Depression	Direct	0.088	0.035	**0.013**
Sugar	Depression	Direct	0.075	0.030	**0.024**
Sugar	DMFT	Direct	0.049	0.020	**0.016**
Sugar	DMFT	Indirect via VPI	0.007	0.003	**0.016**
DMFT	Depression	Total	0.068	0.033	**0.041**
Sex	Sugar	Direct	0.085	0.022	**< 0.001**
Sex	Depression	Direct	0.266	0.030	**< 0.001**

As a sensitivity analysis, we applied inverse probability of treatment weighting (IPTW) including sex, household head’s education, household income, obesity, and visible plaque index in the propensity score model. IPTW results were consistent with the main adjusted Poisson models: high sugar intake was associated with a higher prevalence of depressive symptoms (ATE = 3.96%; 95% CI: 0.79–7.14; IPTW RR = 1.31; 95% CI: 1.06–1.62), but not with caries (Supplementary Table S2).

## Discussion

Our findings revealed a correlation between depression and higher DMFT values in adolescents. This association may be explained by a lower socioeconomic situation, which increases sugar consumption and triggers both conditions mediated by higher sugar consumption.

The correlation between depression and dental caries can be explained by depressed individuals often having poor oral hygiene, which is a predisposing factor for the accumulation of biofilm and, consequently, a greater predisposition to oral diseases [29, 30]. Furthermore, the use of antidepressants and other medications has been linked to hyposalivation and xerostomia, both of which are associated with caries [31]. Additionally, studies indicate that caries lesions can cause aesthetic changes in an individual’s speech and chewing, which can lead to decreased self-esteem, especially in adolescents [32]. Low self-esteem, in some cases, can evolve into depression [33]. In our study, we advanced knowledge by identifying common risk factors for both tooth decay and depression, such as lower socioeconomic indicators and sugar consumption.

The *socioeconomic situation* proved to be a reliable latent variable, with lower values correlating with increased sugar consumption, in line with previous findings [34–36]. One reason for this trend is the affordability and availability of sugar-rich products, along with limited health education, particularly among those with lower educational backgrounds [37, 38].

Lower socioeconomic values also correlated with depression, consistent with findings from prior research [11, 12, 39]. The link between social vulnerability and depression is not fully understood, but untested hypotheses suggest that social vulnerability exposes individuals to early-life stressors [40, 41] and epigenetic inflammatory mechanisms that can arise in fragile environments over the course of life [42].

Moreover, values of lower socioeconomic status were associated with higher DMFT values. These findings are largely consistent with previous studies, which consistently demonstrate an association between social vulnerability and poorer oral outcomes [43, 44]. Apart from predisposing individuals to greater consumption of unhealthy sugary foods, vulnerability can lead to increased cavity rates due to inadequate oral hygiene, limited access to fluoridated water, and challenges in accessing medical and dental care, all of which contribute to an elevated risk of DMFT index [45, 46].

Our findings also revealed that higher consumption of added sugars was linked to depression in adolescents. These results are consistent with previous studies [47–51], including those conducted within this same cohort [14]. The intake of sugars could potentially contribute to mental disorders such as depression, as the sugar present in these foods can disrupt the hypothalamic-pituitary-adrenal axis [52], leading to increased circulation of inflammatory cytokines due to hyperglycemia, thereby promoting inflammation and neuronal degeneration [53–55]. Another mechanism worth mentioning is that sugar intake can alter serotonin receptor uptake [56] and induce neurobiological changes in the reward system, prompting individuals to consume more sugar, potentially leading to substance-related reward disorder and, ultimately, depression [57, 58].

We had already shown that added sugar intake was among the factors most strongly associated with both depression and suicide risk in adolescents from the same population [59]. In addition, the present findings are consistent with a recent investigation within the same São Luís birth cohort, which demonstrated that high free sugar consumption, insulin resistance, and low socioeconomic status are central nodes in a complex network linking caries, sedentary behavior, poor sleep quality, and mental conditions such as depression and suicide risk [60]. The convergence of evidence using different models and analytical approaches within this cohort reinforces the robustness of our results and highlights the role of high sugar intake in both oral and mental health.

Our findings confirm a well-established link between higher sugar consumption and tooth decay [13, 61]. Increased sugar consumption, particularly sucrose, elevates the activity of bacteria such as *Streptococcus mutans*, which produce organic acids and intra- and extracellular polysaccharides. This leads to pH drops and acid production, resulting in demineralization of dental hard tissues. Additionally, it alters bacterial composition, reducing diversity in oral biofilm and promoting the predominance of acidogenic and aciduric bacteria [62, 63].

As secondary outcomes, our study found that girls were more exposed to increased sugar consumption and had higher rates of depression. Numerous studies have already associated women with a higher likelihood of experiencing common mental illnesses compared to men [64, 65]. Female individuals are more prone to facing worse socioeconomic situations, less financial stability, greater food insecurity, inadequate nutritional intake, unstable housing, conflicts with partners, and other factors that impact emotional well-being [66–68]. In terms of dietary habits, women are more inclined to consume foods rich in sugar [69]. This tendency can be attributed to the stress they experience due to the increased workload and hormonal fluctuations during the menstrual cycle, particularly in the luteal phase, which heightens the craving for sugary foods [69].

This study has limitations. Firstly, its cross-sectional design precludes the establishment of temporal relationships between the risk factors for caries and depression. However, the primary objective was to assess an association between these conditions rather than infer causality. Although smoking is a recognized determinant of oral health outcomes, its tax among adolescents in São Luís was 3.56%, according to both our data and national surveys, indicating the city as having the lowest tobacco use in Brazil. This very low prevalence limited its variability and statistical relevance, and its exclusion should be considered when interpreting the findings. As another limitation, unfortunately, individual fluoride exposure and dental attendance/utilization were not systematically available for the entire sample, which limited their inclusion of these covariates in the models. Using this variable could have introduced additional missing data and potential bias; therefore, it was not incorporated into our analytical models.

The findings of this study concern adolescents aged 18 and 19 years in the city of São Luís, Brazil. Therefore, it should be interpreted primarily within this context. Nonetheless, the results may also provide insights for populations of adolescents living in similar social, economic, and epidemiological conditions, particularly in settings marked by social inequities and a high burden of oral diseases and mental health problems.

Among the main strengths of this study is the use of data derived from a population-based sample, which ensures representativeness of adolescents residing in the city of São Luís and enhances the generalizability of the findings. Furthermore, the assessment of depression through the Mini International Neuropsychiatric Interview (M.I.N.I.), a standardized diagnostic tool aligned with the criteria of the fourth version of the Diagnostic and Statistical Manual of Mental Disorders (DSM-IV), provides a robust and clinically validated approach to identifying major and recurrent depressive episodes. This methodological choice minimizes misclassification bias and reinforces the validity of the associations observed. Additionally, the application of Structural Equation Modeling stands out as a significant analytical strength, as it allows for the simultaneous evaluation of complex relationships between observed and latent variables. SEM also contributes to the reduction of measurement errors, offering more precise estimations of the direct and indirect effects of social inequities and sugar consumption on both depression and dental caries.

Our study shows a link between adolescent depression and caries prevalence, likely driven by common risk factors such as low socioeconomic status and high sugar intake. These findings have important implications for both policy and clinical perspectives. Public health interventions that simultaneously address mental health and oral health are needed, particularly in socially vulnerable contexts. In addition, upstream strategies such as taxation and regulatory policies on sugar-rich products, and the incorporation of nutrition and mental health education into school curricula, help reduce the dual burden of depression and dental caries among adolescents. From a clinical standpoint, our results highlight the importance of interdisciplinary collaboration: dental professionals should remain vigilant for psychological distress among adolescents with high caries experience. At the same time, mental health providers should consider oral health as part of comprehensive care. Integrating dentistry and mental health may help mitigate the long-term consequences of depression and oral diseases across the lifespan.

## Supplementary Information

Below is the link to the electronic supplementary material.

Supplementary Material 1 (DOCX 17.9 KB)

## Data Availability

The data that support the findings of this study are available on request from the corresponding author. The data are not publicly available due to privacy or ethical restrictions.
