# Supplementary material for: Depression and caries in adolescents: role of social inequities and sugar consumption
Source: Clin Oral Investig. 2026 Jan 30;30(2):66. doi: 10.1007/s00784-025-06738-y (PMC12855325; doi:10.1007/s00784-025-06738-y)
Supplement: Supplementary file 1 — Supplementary Material 1 (DOCX 17.9 KB) [file 784_2025_6738_MOESM1_ESM.docx]

**Supplementary Table 1.** Sociodemographic and behavioral characteristics of the sample according to depressive symptoms.

| **Variable** | **No depressive symptoms n (%)** | **Depressive symptoms n (%)** |
| --- | --- | --- |
| **Family income, Brazilian monthly minimum wage** |  |  |
| < 1 | 678 (31.62%) | 119 (33.52%) |
| 1 a < 3 | 916 (42.72%) | 163 (45.92%) |
| 3 a < 5 | 297 (13.85%) | 41 (11.55%) |
| ≥ 5 | 253 (11.80%) | 32 (9.01%) |
| **Education of the head of the family** |  |  |
| Illiterate | 25 (1.30%) | 3 (0.94%) |
| Elementary School | 476 (24.77%) | 81 (25.31%) |
| High school | 1,065 (55.41%) | 187 (58.44%) |
| Incomplete higher | 73 (3.80%) | 8 (2.50%) |
| Graduated | 283 (14.72%) | 41 (12.81%) |
| **Adolescent's education** |  |  |
| Elementary School | 55 (2.57%) | 24 (6.76%) |
| High school | 1,509 (70.42%) | 238 (67.04%) |
| Faculty | 579 (27.02%) | 93 (26.20%) |
| **Socioeconomic class** |  |  |
| D/E | 363 (19.17%) | 83 (26.18%) |
| C | 943 (49.79%) | 167 (52.68%) |
| B | 503 (26.56%) | 59 (18.61%) |
| A | 85 (4.49%) | 8 (2.52%) |
| **Sugar intake (acugcat)** |  |  |
| <25 g | 403 (18.80%) | 63 (17.75%) |
| 25–49.9 g | 613 (28.59%) | 91 (25.63%) |
| 50–74.9 g | 454 (21.18%) | 72 (20.28%) |
| ≥75 g | 674 (31.44%) | 129 (36.34%) |
| **Visible plaque index (IPV)** |  |  |
| <15% | 818 (40.34%) | 129 (37.94%) |
| ≥15% | 1,210 (59.66%) | 211 (62.06%) |

**Supplementary Table 2**. Association between high sugar intake and mental and oral health outcomes in adolescents using Inverse Probability of Treatment Weighting (IPTW) as a Sensitivity Analysis.

| **Outcome** | **N analyzed** | **ATE (Risk Difference) (95% CI)** | **p-value** | **IPTW Risk Ratio (95% CI)** | **p-value** |
| --- | --- | --- | --- | --- | --- |
| **Depression** | 2,114 | 0.0396  (0.0079 – 0.0714) | 0.014 | 1.31  (1.06 – 1.62) | 0.013 |
| **Caries** | 2,071 | 0.0187  (−0.0154 – 0.0528) | 0.283 | 1.02  (0.98 – 1.07) | 0.294 |
